# Identifying Flare Prone Spondyloarthritis: Insights From a Prospective Cohort

**DOI:** 10.7759/cureus.107675

**Published:** 2026-04-24

**Authors:** Abilash Krishnan V, Sayan Mukherjee, Mukesh Kumar Maurya, Urmila Dhakad, Nishant Kamble, Ankush PM, Aditi Singh, Puneet Kumar

**Affiliations:** 1 Clinical Immunology and Rheumatology, King George's Medical University, Lucknow, IND; 2 Rheumatology, King George's Medical University, Lucknow, IND; 3 General Medicine, Era's Lucknow Medical College and Hospital, Lucknow, IND; 4 Medicine, King George’s Medical University, Lucknow, IND

**Keywords:** antirheumatic agents, axial spondyloarthritis, drug tapering, mathematical models, symptom flare up

## Abstract

Background

Flares are episodes of disease worsening requiring a change in treatment. Incidence of flares in spondyloarthritis (SpA) is around 60% in longitudinal patient-reported studies. The study was conducted to determine characteristics of flares in SpA and to identify differences between flare and non-flare populations. We hypothesized that the patient cohort who develop flares may behave differently in terms of validated disease activity and functional indices with regard to the non-flare population.

Methods

A cohort of 106 patients (2650 patient-weeks follow-up and 318 visits) who fulfilled the European Spondyloarthropathy Study Group (ESSG) or Assessment of SpondyloArthritis International Society (ASAS) classification criteria for SpA were followed up for six months. Diagnosis of flare was made by the rheumatologist using patient-reported indices and ruling out confounders. Statistical methods for analysis include the Mann-Whitney U test, ANOVA, chi-square, survival analyses, and longitudinal analyses by mixed-effects models, repeated measures ANOVA.

Results

At six months, 67 of 106 patients (63.2%) developed flares, of whom 51 (76.1%) had major (generalized) flares, and 16 (23.9%) had minor (localized) flares. In univariable analyses, shorter disease duration (<6 years), baseline enthesitis, baseline glucocorticoid use, and higher baseline disease activity were associated with increased odds of flare, whereas radiographic axial SpA was associated with reduced odds. In multivariable logistic regression analysis, inactive disease (OR: 0.21; 95% CI: 0.05-0.94; p = 0.042) and low disease activity at baseline (OR: 0.25; 95% CI: 0.09-0.72; p = 0.011) were independently associated with lower odds of flare, while baseline enthesitis was associated with higher odds (OR: 11.29; 95% CI: 1.30-93.46; p = 0.025). The median time to flare was longer in patients receiving biologic or targeted synthetic DMARDs (88 days vs 26 days), although this difference did not reach statistical significance (p = 0.249). Longitudinal analyses using general linear and mixed-effects models demonstrated significant differences in trajectories of ASAS-validated indices between flare and non-flare groups.

Conclusion

Baseline disease activity and enthesitis status are key determinants of flare risk in SpA. Patients with inactive or low disease activity have lower odds of flare, whereas the presence of enthesitis is associated with increased risk.

## Introduction

Spondyloarthritis (SpA) encompasses a spectrum of chronic inflammatory disorders primarily affecting the spine and sacroiliac joints, often accompanied by enthesitis involving the axial and peripheral skeleton. Disease flares represent episodes of clinically meaningful worsening that necessitate therapeutic modification and are associated with adverse outcomes [1,2]. Definitions of flares are diverse, owing to the multifaceted nature of the disease. The Assessment of SpondyloArthritis International Society (ASAS) expert group evaluated multiple definitions of clinically important worsening and recommended an increase in the Ankylosing Spondylitis Disease Activity Score using C-reactive protein (ASDAS-CRP) of ≥0.9 as a criterion for clinically important deterioration [3]. A study assessing outcome measures to detect flares identified absolute change in Bath Ankylosing Spondylitis Disease Activity Index (BASDAI) of more than or equal to 2 or a relative change of ≥30% in subcomponents of BASDAI to indicate a meaningful symptomatic deterioration [4]. Agreement between the perception of flares by doctors and patients was not high (Cohen’s kappa coefficient of 0.61), which indicated a lacuna in the diagnosis of flares [5]. Another study divided flares into generalized/major and localized/minor flares. Minor flares were pain/swelling restricted to one area with fatigue and stiffness, and major flares were "generalized pain, hot burning joints, muscle spasm, fever, sweating, extreme fatigue, and stiffness"[6].

Evidence from tumor necrosis factor inhibitor (TNFi) discontinuation trials demonstrates high flare rates following withdrawal, with reported rates approaching 79%. Long-term follow-up data (e.g., ESTHER trial) indicate comparable flare rates after discontinuation across treatment groups, suggesting limited sustained protective effect after cessation [7]. Conversely, ongoing TNFi therapy or re-initiation is associated with reduced flare incidence [8,9]. Despite their efficacy, prolonged TNFi use is often limited in low- and middle-income settings due to cost constraints, making dose tapering and interval extension common clinical strategies [10]. Given these considerations, identifying predictors of flare is clinically relevant for individualized treatment planning. This study aimed to characterize flare patterns and determine predictors of flare in SpA over a six-month follow-up period using validated ASAS indices. We hypothesized that patients who develop flares exhibit distinct longitudinal trajectories in disease activity and functional indices compared to those who remain flare-free. A preprint of this manuscript is available online [11].

## Materials and methods

Study design and participants

**Figure 1:**
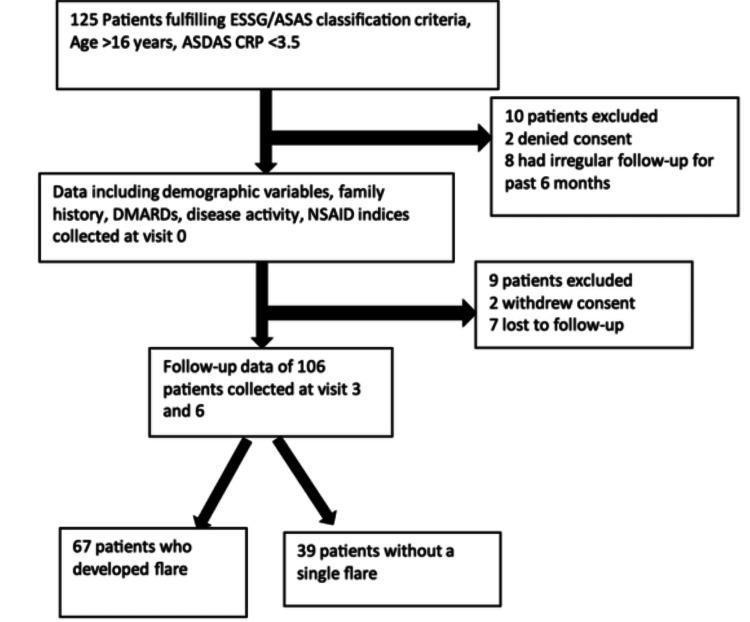
Flow diagram of the study.

This observational longitudinal cohort study was conducted at the Department of Clinical Immunology and Rheumatology in a tertiary care teaching hospital in North India between 2021 and 2023. Adult patients (≥18 years) fulfilling the European Spondyloarthropathy Study Group (ESSG) or ASAS classification criteria for SpA were eligible [12]. Participants were required to have at least six months of prior follow-up at the study center. Patients with very high disease activity at baseline and pregnant individuals were excluded. Exclusion of patients with very high baseline disease activity was undertaken to ensure reliable identification of subsequent flares using validated indices. The study was approved by the Institutional Ethics Committee of King George’s Medical University (Ref: VII PGTSC-IIA/P14), and informed written consent was obtained from all participants (Figure 1).

Sample

Sample size estimation was done using the formula for sample size for comparing two proportions, where we assume the proportion in untreated group 1 (P1) = 0.90, proportion in treated group 2 (P2) = 0.70, significance level (α) = 0.05, and Power (1 - β) = 0.8. The sample size for comparing two proportions was calculated using:

\begin{equation}

n = \frac{\left[ Z_{1-\alpha/2}\sqrt{2P(1-P)} + Z_{1-\beta}\sqrt{P_1(1-P_1) + P_2(1-P_2)} \right]^2}{(P_1 - P_2)^2}

\end{equation}

The sample size was further adjusted for repeated measurements using the design effect:

\begin{equation}

n_{\text{adjusted}} = \frac{n}{1 + (m - 1)\rho}

\end{equation}

where $m$ represents the number of repeated measurements and $\rho$ denotes the intra-class correlation coefficient. This yielded a requirement of 62 participants per group. After adjustment for repeated measures (baseline, three months, and six months), the effective sample size was 33 participants per group.

Data collection

Baseline and follow-up data (at three and six months) included demographic characteristics, disease duration, and validated disease activity measures: ASDAS-CRP, BASDAI, Maastricht Ankylosing Spondylitis Enthesitis Score (MASES), tender and swollen joint counts, Bath Ankylosing Spondylitis Functional Index (BASFI), Bath Ankylosing Spondylitis Metrology Index (BASMI), and ASAS NSAID index.

Disease activity was categorized using ASDAS-CRP thresholds: inactive (<1.3), low (1.3≤2.1), high (2.1≤3.5), and very high (≥3.5).

Clinical variables collected included prior flares, medication changes, infections, treatment adherence, and extra-articular manifestations. Drug inadequate response (DMARD-IR) was determined by the treating rheumatologist based on patient's global assessment and lack of ASAS-defined improvement. The data consisted of measures enlisted in the ASAS coreset for clinical record keeping.

Diagnosis of flare

A flare was defined as clinically significant worsening requiring treatment escalation after study initiation.

Flares were classified as:

Localized (minor): involvement of a single site without systemic features

Generalized (major): multi-site involvement and/or axial symptom worsening with systemic features, accompanied by an increase in ASDAS-CRP ≥ 0.9

Flare subtypes included axial, peripheral arthritis, enthesitis, mixed patterns, and extra-articular manifestations (e.g., uveitis) [13]. Diagnoses were confirmed by the treating physician after exclusion of alternative causes, including infection, trauma, and metabolic conditions.

**Figure 2:**
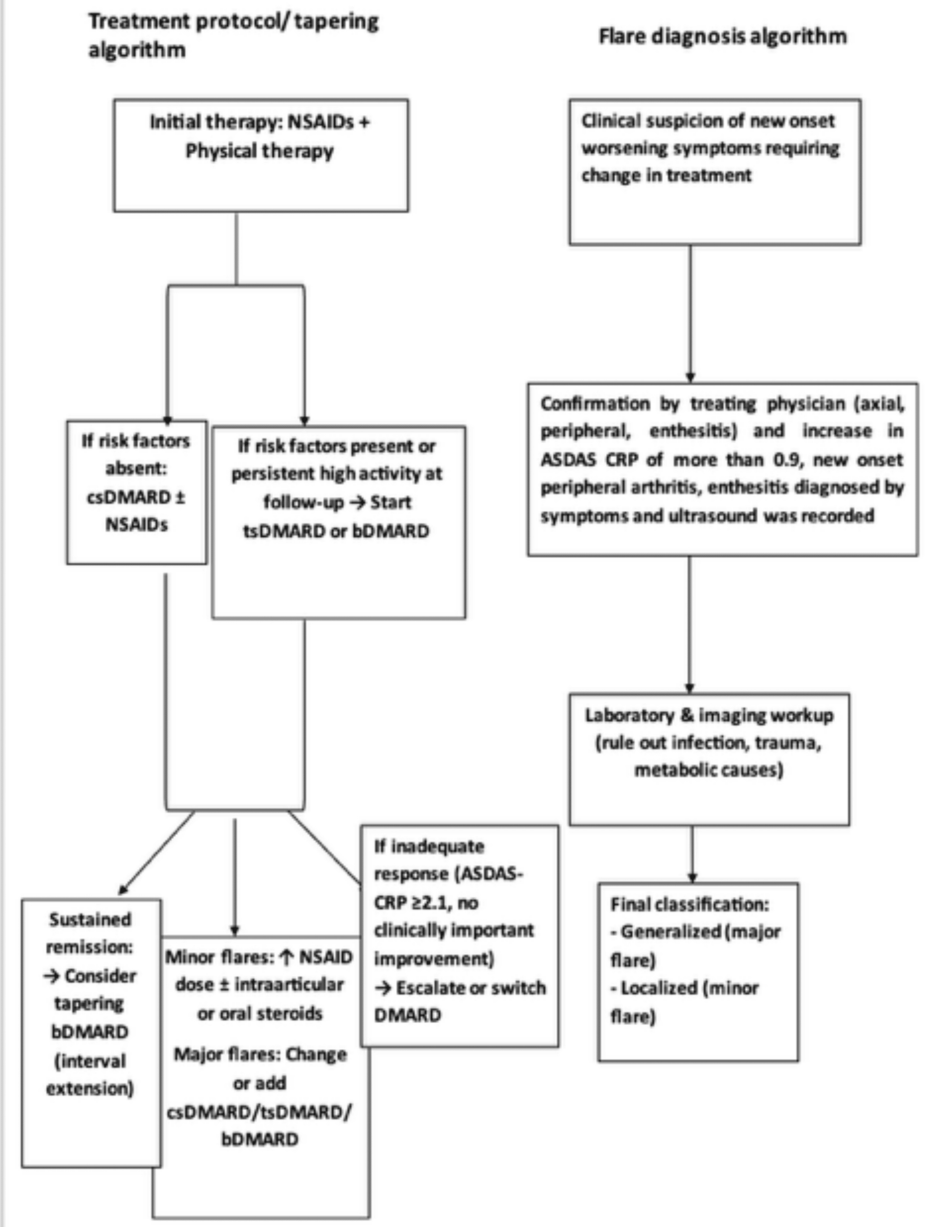
Treatment protocol and flare diagnosis algorithm.

Objective confirmation of enthesitis and synovitis was performed using ultrasound according to OMERACT definitions [14]. Laboratory investigations include a complete blood count, erythrocyte sedimentation rate (ESR in mm/hour), CRP and workup for infections (including blood, urine, and sputum culture and sensitivity, routine stool testing) depending on the presentation (Figure 2).

Details of Treatment

All patients received non-steroidal anti-inflammatory drug (NSAIDs) and structured physical therapy as first-line management. The use of conventional synthetic DMARDs (csDMARDs), biologic DMARDs (bDMARDs), and targeted synthetic DMARDs (tsDMARDs) was guided by disease severity, prognostic factors, patient preference, and financial considerations. Due to cost constraints, bDMARDs were preferentially used in patients with poor prognostic features or inadequate response to csDMARDs. Tapering of bDMARDs was performed through interval extension following achievement of sustained inactive disease, in accordance with ASAS-EULAR recommendations.

Treatment of Flares

Most localized flares were treated with increasing dose and frequency of NSAIDs. Some patients also required intraarticular or brief oral glucocorticoids. Depending upon the duration and severity of major flares, change/addition of therapy was considered by the treating team.

Statistical Analysis

Descriptive data were expressed as median (due to non-normality of data), inter-quartile range (IQR), frequency, and proportions. Comparative analyses between patients who developed and who did not develop flares at six months were performed. Data normality was assessed using the Shapiro-Wilk test. Based on the distribution, the Mann-Whitney U test was used for non-normally distributed data, while ANOVA was applied for normally distributed data. Categorical variables were analyzed using the chi-squared test.

Univariable Analysis

We first conducted univariable logistic regression analyses to assess the association between each candidate predictor and the outcome. These variables include demographic factors (age, sex, duration of disease, and family history), disease classification (radiographic vs non-radiographic SpA), known risk factors for progression (presence of syndesmophytes, bDMARDs at baseline, bDMARD inadequate response prior to enrolment, steroid requirement at baseline, ASAS NSAID index, hip involvement, current or past enthesitis, and disease activity at baseline). The purpose of this initial analysis was to screen variables for potential inclusion in the multivariable model. Variables with a p-value <0.10 in the univariable analysis were considered for the next stage. This threshold was intentionally liberal to reduce the risk of omitting potentially important predictors.

Stepwise Selection for Multivariable Analysis

From the pool of candidate variables identified above, we applied a manual backward stepwise selection approach. Starting with a full model including all eligible variables from the univariable analysis, we iteratively removed variables based on their statistical insignificance (p > 0.05) and lack of substantial improvement to model fit (evaluated using likelihood ratio tests). Variables known to be clinically relevant or previously established in literature (e.g., age, sex, and presence of syndesmophytes) were retained regardless of p-value to preserve clinical interpretability and avoid model misspecification. Variables related to treatment dosage and type of bDMARDs were not included in the analysis due to heterogeneity in treatment regimens and lack of uniform data, which would have introduced significant confounding and potential bias. We assessed the final model for multicollinearity (using variance inflation factor) and goodness-of-fit (Hosmer-Lemeshow test).

A general linear statistical model for repeated measures analysis was done to assess longitudinal change in ASAS-validated indices between flare and non-flare group. The Kaplan-Meier survival analysis was performed to compare the time to flare between patients who were taking/not taking biological or targeted synthetic DMARDs (b/tsDMARDs) at baseline. The event was defined as the occurrence of a disease flare, confirmed by the treating physician based on clinical and laboratory parameters necessitating treatment escalation. Patients without a documented flare during follow-up were censored at their last recorded visit. The time origin was the date of baseline assessment. Also, the Kaplan-Meier survival analysis was performed to compare flare-free survival between inactive, low, and high disease activity patients at baseline.

## Results

Baseline variables

The median age of our population was 26 years (IQR: 22-31), and the median duration since diagnosis was 4.5 years (IQR: 3-8). A total of 10 (9.4%) patients had a family history of SpA, and all of them were first-degree relatives. There were 85 (80.1%) males and 21 (19.9%) females enrolled in the study, with a male: female ratio of roughly 4:1. A total of 75 (70.8%) patients had radiographic SpA, whereas 31 (29.2%) had nr-SpA. Among 69 patients tested for HLA-B27, 50 (72.5%) were positive and 19 (27.5%) were negative. HLA-B27 status was unknown in 37 patients (34.9% of the total cohort). Seventy-two (68%) patients had SpA with axial and peripheral involvement, 31 (29.2%) had pure axial SpA, 2 (1.9%) had pure peripheral SpA, and 1 (0.9%) had IBD-associated arthritis. A total of 50 (47.2%) patients had syndesmophytes at baseline, and 31 (29.2%) patients had root joint (hip/shoulder) disease at baseline. A total of 24 (22.6%) patients had a current/past history of enthesitis, and 14(13.1%) patients had a current/past history of uveitis at baseline. A total of 84 (79.2%) were on csDMARDs, and 23 (21.7%) patients were on bDMARDs at baseline. A total of 48 (45.2%) patients did not have a fall of ASDAS-CRP of > 1.1 and a significant fall in pain Visual Analog Scale (VAS) (did not achieve pain VAS <4) on adequate trial of full dose csDMARDs prior to enrolment, and 9 (8.5%) patients failed at least 1 bDMARD/tsDMARD prior to enrolment. Patients were declared bDMARD/tsDMARD IR after receiving an adequate trial of optimal dose bDMARDs/tsDMARDs. Thirteen (12.3%) patients required oral glucocorticoids at baseline (Table 1).

**Table 1:** Baseline demographic, clinical, and treatment characteristics of the patient cohort (n = 106). ASAS: Assessment of SpondyloArthritis International Society; ASDAS: Ankylosing Spondylitis Disease Activity Score; BASDAI: Bath Ankylosing Spondylitis Disease Activity Index; BASFI: Bath Ankylosing Spondylitis Functional Index; BASMI: Bath Ankylosing Spondylitis Metrology Index; bDMARD: biologic DMARD; CRP: C-reactive protein; csDMARD: conventional synthetic DMARD; ESR: erythrocyte sedimentation rate; MTX: methotrexate; NSAID: non-steroidal anti-inflammatory drug; SSZ: sulfasalazine; tsDMARD: targeted synthetic DMARD; VAS: Visual Analog Scale; SpA: spondyloarthritis.

Variable	Value
Age in years, median (IQR)	26 (22-31)
Duration of disease in years, median (IQR)	4.5 (3-8)
Male, n (%)	85 (80.1)
Family history of SpA, n (%)	10 (9.4)
Presence of syndesmophytes, n (%)	50 (47.2)
Radiographic SpA, n (%)	75 (70.8)
Non-radiographic SpA, n (%)	31 (29.2)
HLA-B27 status available, n (%)	69 (65)
Positive	50 (72.5)
Negative	19 (27.5)
Pattern n (%): Axial + Peripheral SpA	72 (69)
Peripheral SpA	2 (1.9)
IBD - arthritis	1 (0.9)
Axial SpA	31 (29.2)
Hip involvement	31 (29.2)
Enthesitis	24 (22.6)
Uveitis	14 (13.2)
ASAS-NSAID index, median (IQR)	25 (1-100)
ESR in mm/hour, median (IQR)	25 (14-40)
CRP in mg/l, median (IQR)	6.85 (3-16.9)
BASDAI, median (IQR)	2.4 (1.2-3.6)
ASDAS-CRP, median (IQR)	2.5 (1.8-3.2)
BASFI, median (IQR)	2.2 (0.7-3.4)
BASMI, median (IQR)	1.8 (1.2-2.6)
Systemic glucocorticoid use at baseline, n (%)	13 (12.3)
Median dose of glucocorticoid	5 mg/day prednisolone equivalent
SSZ only, n (%)	44 (41.5)
MTX only, n (%)	22 (20.8)
SSZ + MTX, n (%)	18 (17)
Adalimumab Q2W, n (%)	9 (8.5)
Adalimumab Q3W, n (%)	6 (5.7)
Adalimumab Q6W, n (%)	3 (2.8)
Infliximab Q3M, n (%)	1 (0.9)
Secukinumab monthly, n (%)	4 (3.8)
Tofacitinib, n (%)	8 (7.5)

Characteristics of flares

Sixty-seven patients (63.2%) developed flare during the course of the study period. Nine different patterns of flare have been identified in the study, namely pure axial flare (17%), axial and peripheral flare (15%), minor enthesitis flare (8%), major arthritis flare (6%), major enthesitis flare (5%), minor arthritis flare (4%), axial and entheseal flare (4%), uveitis (4%), and cutaneous flare (1%). Of the 67 patients who flared, associations of flare were history of drug default in 20 (30%) patients, antecedent infections in 13 (19%) patients (eight patients had viral upper respiratory tract infection, three patients had urinary tract infections, and two patients had acute gastroenteritis), and unknown cause in 34 (51%) patients.

Qualitative variables were analyzed and significant association favoring flare was seen with duration since diagnosis of less than six years (unadjusted OR: 2.17 (95% CI: 0.97-4.86)), steroid requirement at baseline (unadjusted OR: 8.29 (95% CI: 1.03-66.46)), current/past history of enthesitis at baseline (unadjusted OR: 5.48 (95% CI: 1.51-19.82)) and disease activity at baseline (p = 0.002) while radiographic SpA (unadjusted OR: 0.31 (95% CI: 0.11-0.83)) was associated with lower tendency of flare (Table 2).

**Table 2:** Distribution of qualitative variables between flare and non-flare groups. # Inactive disease was defined as ASDAS < 1.3, low disease activity as 1.3≤2.1, high disease activity as 2.1≤3.5, and very high disease activity as ≥3.5. Categorical variables are presented as a number (%). ASDAS: Ankylosing Spondylitis Disease Activity Score; bDMARD: biologic DMARD; tsDMARD: targeted synthetic DMARD.

Variables		Frequency (%)		p-value
		Flare n = 67 (63.2)	Non-flare n = 39 (36.8)	
Duration of disease	Six years or less	49 (73.1)	21 (53.8)	0.043
	More than six years	18 (26.8)	18 (46.1)	
Sex	Male	50 (74.6)	35 (89.7)	0.078
	Female	17 (25.4)	4 (10.2)	
Radiographic		42 (62.6)	33 (84.6)	0.017
Non-radiographic		25 (37.3)	6 (15.3)	
Presence of syndesmophytes at baseline		30 (44.7)	20 (51.2)	0.518
Family h/0		4 (5.9)	6 (15.3)	0.110
bDMARDs/tsDMARDs at baseline		35 (52.2)	16 (41)	0.265
Steroid req at baseline		12 (17.8)	1 (2.6)	0.029
Age group	<25	32 (47.7)	20 (51.2)	0.777
	25-35	25 (37.3)	12 (30.7)	
	>35	10 (14.9)	7 (17.9)	
Hip involvement		18 (26.8)	13 (33.3)	0.480
bDMARD inadequate response prior to enrolment		6 (8.9)	3 (7.6)	1.000
Current/past history of enthesitis		21 (31.3)	3 (7.6)	0.007
Disease activity at baseline^#^	Inactive	3 (4.4)	7 (17.9)	0.002
	Low	11 (16.4)	14 (35.9)	
	High	53 (79.1)	18 (46.1)	

Disease duration, when modeled as a continuous variable in a Cox proportional hazards model, was not significantly associated with time to flare (HR = 0.95; 95% CI: 0.89-1.02; p = 0.171). This differs from the dichotomized model (<6 years vs ≥6 years), where shorter disease duration showed a higher unadjusted risk of flare (OR = 2.17; 95% CI: 0.97-4.86). The difference likely reflects the loss of information due to dichotomization or may reflect the differences in disease behavior. Multivariable logistic regression analyses revealed inactive (adjusted OR: 0.21 (95% CI: 0.05-0.94); p = 0.042) or low disease at baseline (adjusted OR: 0.25 (95% CI: 0.09-0.72); p = 0.011) was significantly associated with lower tendency of flare while current/past enthesitis at baseline (adjusted OR: 11.29 (95% CI: 1.30-93.46); p = 0.025) favored greater tendency to flare (Table 3).

**Table 3:** Univariable and multivariable logistic regression analysis to compare flare and non-flare groups. Note: High disease activity serves as the reference category. Multivariable logistic regression adjusted for age, sex, disease duration, HLA-B27 status, and use of b/tsDMARDs at baseline. Greater disease activity is indicated by higher ASDAS and BASDAI scores. A positive odds ratio represents a higher likelihood of flare occurrence. When sensitivity analysis was done for disease duration as a continuous variable, Cox proportional hazards model did not show a significant difference (HR = 0.95; 95% CI: 0.89-1.02; p = 0.171). *Disease activity at baseline showed significant association with tendency to flare whereas, the adjusted odds ratio of low/inactive disease developing a flare is indicated in subsequent rows. NA: not applicable; ASDAS: Ankylosing Spondylitis Disease Activity Score; BASDAI: Bath Ankylosing Spondylitis Disease Activity Index; bDMARDs: biologic DMARDs; tsDMARDs: targeted synthetic DMARDs; SpA: spondyloarthritis.

Variables	Univariable analysis			Multivariable analysis			
	Unadjusted odds ratio	95% CI	p-value	Adjusted odds ratio	95% CI	p-value	
Male sex	0.34	0.10-1.08	0.060	0.28	0.08-1.03	0.055	
Duration less than six years	2.17	0.97-4.86	0.043	2.77	0.86-8.99	0.089	
Radiographic SpA	0.31	0.11-0.83	0.017	0.52	0.16-1.66	0.269	
Glucocorticoid requirement at baseline	8.29	1.03-66.46	0.020	3.95	0.42-37.05	0.229	
Disease activity at baseline*	NA	NA	0.002	NA	NA	0.011	
Inactive disease vs high at baseline	NA	NA	NA	0.21	0.05-0.94	0.042	
Low disease vs high at baseline	NA	NA	NA	0.25	0.09-0.72	0.011	
Current/past enthesitis at baseline	5.48	1.51-19.82	0.005	11.29	1.30-93.46	0.025	

*Change of ASAS-Validated* *Indices in Flare and Non-flare Groups*

The longitudinal change in quantitative variables at 0, 3, and 6 months was compared as individual medians at different time-points. A general linear model for repeated measures (two-way ANOVA) revealed that the longitudinal change in means of ASDAS-CRP, BASDAI, BASFI, BASMI, ASAS-NSAID Index, and Patient General Assessment (PGA) VAS was statistically significant in both intra-group comparison and between flare and non-flare groups at the three time-points. However, with both time and flare outcome as a combined function, only ASDAS-CRP and BASDAI was statistically significant in multivariate tests (Table 4). A mixed-effects model determined that between-groups b and 95% CI were significantly different between flare and non-flare populations.

**Table 4:** General linear model for repeated measures to assess significance of longitudinal change in indices between flare and non-flare groups and within these groups. ASAS: Assessment of SpondyloArthritis International Society; ASDAS: Ankylosing Spondylitis Disease Activity Score; BASDAI: Bath Ankylosing Spondylitis Disease Activity Index; BASFI: Bath Ankylosing Spondylitis Functional Index; BASMI: Bath Ankylosing Spondylitis Metrology; CRP: C-reactive protein; VAS: Visual Analog Scale; NSAID: non-steroidal anti-inflammatory drug.

Dependent variables		Mean at 0, 3, and 6 months, respectively		Intra-group comparison (sig.)		Inter-group comparison (sig.)	Multivariate tests (Wilks’ Lambda)	
		Flare	No flare	Time	Time*flare outcome	Flare outcome	Time	Time*flare outcome
ASDAS-CRP	0	2.70	1.90	0.000	0.030	0.000	0.000	0.015
	3	2.34	1.76					
	6	1.88	1.66					
BASDAI	0	3.02	1.43	0.000	0.034	0.000	0.000	0.012
	3	2.46	1.25					
	6	1.77	0.97					
ASAS-NSAID Index	0	49.90	30.60	0.000	0.321	0.000	0.000	0.228
	3	44.20	15.90					
	6	21.60	4.20					
BASMI	0	2.20	1.88	0.000	0.609	0.334	0.000	0.668
	3	1.92	1.70					
	6	1.77	1.51					
BASFI	0	3.29	1.64	0.018	0.187	0.026	0.000	0.144
	3	2.01	1.36					
	6	1.47	1.07					
Patient General Assessment VAS	0	3.94	2.49	0.001	0.537	0.000	0.000	0.458
	3	3.70	2.15					
	6	2.80	1.70					

Survival Analysis

**Figure 3:**
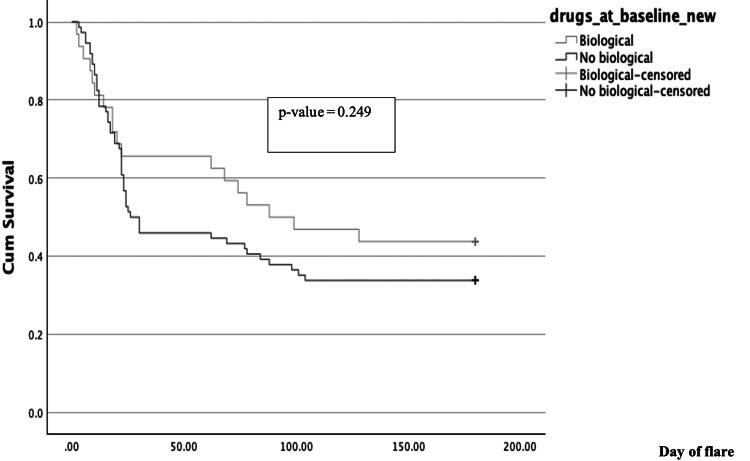
Kaplan-Meier survival analysis to compare time to flare between patients on bDMARDs/tsDMARDs and not on bDMARDs/tsDMARDS. bDMARDs and tsDMARDs have been clubbed together in this survival curve. Patients who received biologicals/tsDMARDs were quite heterogenous, with patients receiving TNFi, secukinumab, and tofacitinib, many receiving tapering doses. bDMARDs: biologic DMARDs; tsDMARDs: targeted synthetic DMARDs; TNFi: tumor necrosis factor inhibitors.

Survival analysis using the Kaplan-Meier analysis showed the median time to flare in the biological/targeted synthetic DMARD group to be at 88 days compared to 26 days in the group without biological/targeted synthetic DMARDs at baseline. This did not meet statistical difference (p = 0.249). The difference in the probability of developing a flare did not meet statistical significance because most patients were on suboptimal/tapering doses of bDMARDs (Figure 3).

**Figure 4:**
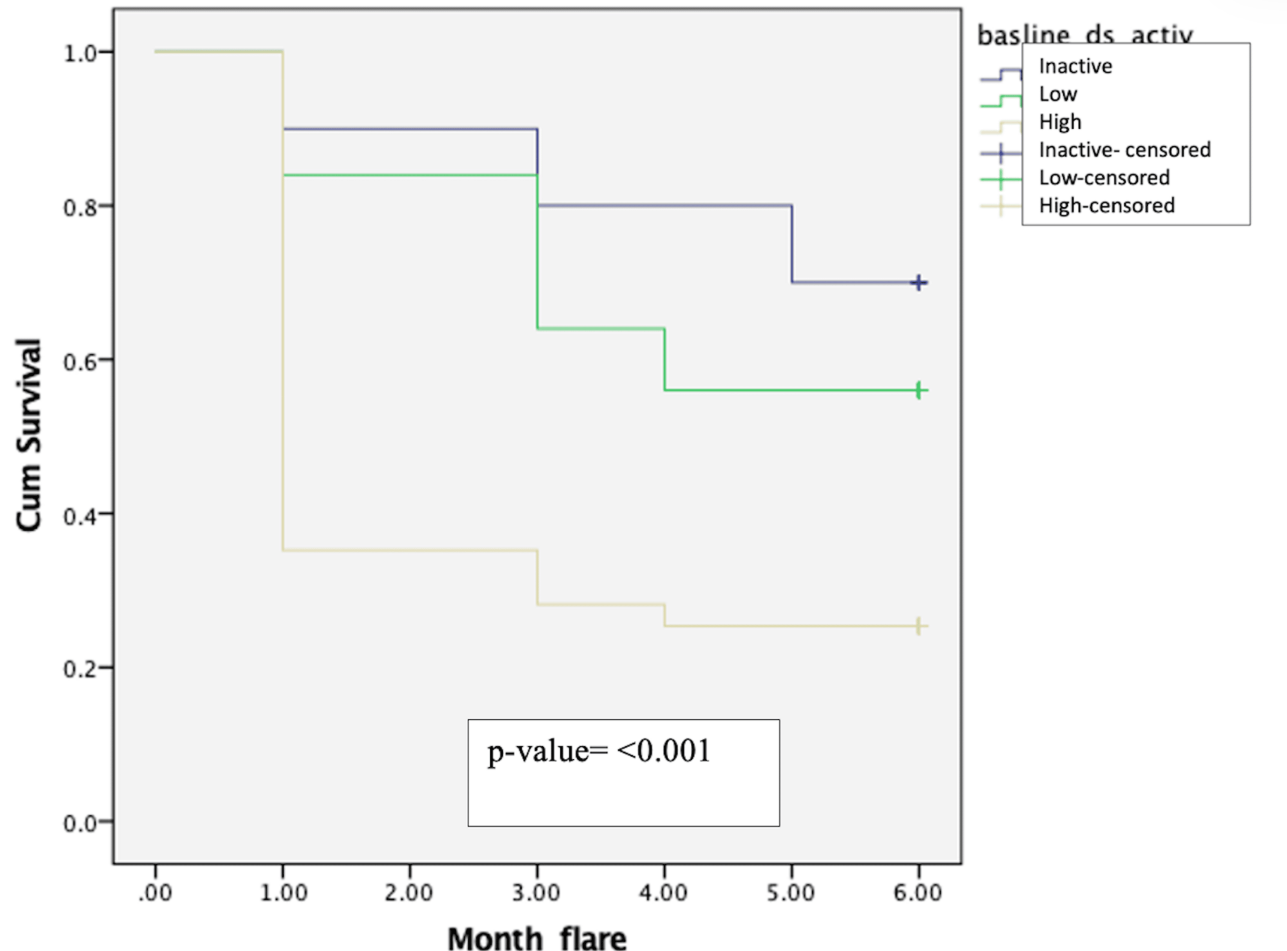
Kaplan-Meier survival curve comparing flare-free survival between inactive, low, and high baseline disease activity groups.

Also, the Kaplan-Meier survival analysis between inactive, low, and high disease activity patients at baseline showed median time to develop flare was 153 days in the baseline inactive disease group and 133.2 days in the baseline low disease group compared to 74.7 days in the baseline high disease group. The difference noted was statistically significant (p < 0.001) (Figure 4).

## Discussion

The study aimed at exploring the characteristics of flares, the relation of disease activity indices to flare episodes, and factors affecting flares. Most of the studies on flares in SpA were conducted on patient-reported flare using multiple methods, namely online questionnaire [6], smartphone app [15], patients’ self-recorded details of NSAID intake and disease activity, and online survey platform [16-20]. The study focused on rheumatologist-diagnosed flares with patient-based indices (BASDAI, ASDAS-CRP, ASAS-NSAID index, patient global assessment VAS, and BASFI) to make the diagnosis of flare. We augmented the patient’s perception of disease activity with a rheumatologist assessment to bridge the doctor-patient consensus in the diagnosis of flare. Also, we relied on ASDAS-CRP, a weighted measure, which considers the burden of inflammation. The details were collected through face-to-face interaction, which further helped in therapeutic decision-making and effective control of flares. Compared to studies on flares in SpA, our cohort was younger and had a relatively shorter disease course (median duration of disease in our population was 4.5 years, with an IQR of 3-8, and a median age of our population was 26, with an IQR of 22-31). Also, the lower median age in our sample population corresponds to the demographic characteristics of the Indian population [6]. The incidence of flares in our study is 63.2%. The history of drug default was as high as 30% in the past month, and antecedent infections were reported 19% of patients in the past three weeks (19%) in the flare population. These associations are considerable challenges in managing chronic diseases in low-to middle-income countries. The reasons for drug default and the causes of infections need further study. The flare incidence was relatively low in the study compared to other SpA cohorts.

Almost all our patients were on NSAIDs, except four patients (3.8%) who did not require NSAIDs during the study. Eighty-four patients (79.3%) were on csDMARDs, and 29.2% were on tapering/regular course bDMARDs/tsDMARDs in our study. Survival analysis showed the median time to flare in the biological group to be 88 days compared to 26 days in the group with no biologicals at baseline, but this did not meet statistical significance. Also, the incidence of flares between the groups on bDMARDs/tsDMARDs and not on bDMARDs/tsDMARDs did not meet a statistically significant difference (33% vs 30.2%). Among patients on bDMARDs, 31.2% of our patients were on tapering doses of bDMARDs, explaining the lack of difference in incidence of flares in patients receiving bDMARDs. None of the patients on tsDMARDs were on inadequate/tapering doses. Similarly, comparative analysis of sulfasalazine-treated and etanercept-treated patients from the ESTHER trial also showed that flare rate was similar in both groups at 24.4 weeks after discontinuation (69% vs 75%) [7]. A study conducted in South India showed that short-course infliximab followed by continuation of methotrexate and sulfasalazine combination can prolong time to disease flare and decrease requirement for additional infliximab dose in SpA. Also, disease flare occurred only in 33.3% (15/45) of patients after a mean duration of 14.5 months compared to the usual time to flare of four to six months on discontinuation of TNFi in the study [21]. There is a need to further evaluate the dynamics of drugs or drug combinations that prevent or reduce the incidence of flares in SpA.

Significant association favoring flare was seen with duration since diagnosis of less than six years, steroid requirement at baseline, a current/past history of enthesitis at baseline, high disease activity at baseline, and radiographic SpA were associated with lower tendency to flare in univariable analyses. Jacquemin et al. also identified significant statistical association of shorter time since onset of disease, higher BASDAI, less anti-TNF treatment, and worse quality of life favoring tendency to flare [18]. Presence of high baseline disease activity has been consistently associated with increased tendency to flare in other longitudinal cohorts too [6] [20]. The results of change in ASDAS-CRP, BASDAI, BASFI, and ASAS-NSAID index at six months was similar to study by Danda et al., which showed higher fall in peripheral arthritis group with similarly high BASDAI, BASFI, ASDAS-CRP, and ASAS-NSAID index at baseline [22]. 

Limitations include a single-center study and a limited sample population preventing in-depth analyses regarding effect of drugs/drug combinations on flare outcomes. Also, the study did not include indices for assessing quality of life and work productivity in relation to flares. The treatment protocol followed in the study does not reflect standard guidelines in SpA, mainly due to financial concerns precluding the use of bDMARDs early in the course of the disease and institutional experience of using csDMARDs. The study was conducted in a single North Indian tertiary care cohort, making it difficult to generalize the findings. Additionally, the study design may limit the ability to control for confounders related to the timing of flares. The study cohort was not powered to conduct subgroup analyses in detail. Strengths of the study include 2650 patient-weeks of follow-up and 318 patient visits, with close patient interaction and tracking of flares. In addition, the study provides real-world data regarding the status of bDMARD/csDMARD use in populations not affording continuous use of bDMARDs due to out-of-pocket health expenditure. Patients with risk factors/protective factors toward flare can be identified, and suitable decisions regarding care plans can be taken with the findings generated by the study.

## Conclusions

Baseline disease activity and enthesitis status are key determinants of flare risk in SpA. Patients with inactive or low disease activity have lower odds of flare, whereas the presence of enthesitis is associated with an increased risk. ASDAS-CRP, BASDAI, PGA VAS, and ASAS-NSAID scores are significantly different in patients of SpA with higher odds of flare. These findings may inform risk stratification and guide individualized treatment strategies.
